# Importance of Ultraviolet-C (UV-C) Emitter Configuration for Clostridioides difficile Attenuation

**DOI:** 10.7759/cureus.71096

**Published:** 2024-10-08

**Authors:** Carmen T Brindeiro, Franklin Dexter, Michelle C Parra, Kaitlin M Walker, Soyun M Hwang, Brendan T Wanta, Debra J Szeluga, Brent A Hadder, Melinda S Seering, Jonathan E Charnin, Randy W Loftus

**Affiliations:** 1 Laboratory Science, RDB Bioinformatics, Coralville, USA; 2 Anesthesia, University of Iowa, Iowa City, USA; 3 Anesthesiology and Perioperative Medicine, Mayo Clinic, Rochester, USA; 4 Microbiology, RDB Bioinformatics, Coralville, USA; 5 Anesthesiology, Mayo Clinic, Rochester, USA

**Keywords:** efficacy, emitter, emitter configuration, ultraviolet-c, uv-c

## Abstract

Background: The impact of ultraviolet-C (UV-C) emitter configuration on pathogen attenuation has not been assessed. We hypothesized that emitter configuration would impact UV-C efficacy for *Clostridioides difficile *(*C. difficile*) attenuation.

Methods: *C. difficile* carriers (ReadyNow^TM^ Test Carriers, Stratix Labs Corporation, Saint Paul, MN) inoculated with > 10^8 ^*C. difficile* American Type Culture Collection (ATCC) 43593 (according to American Society for Testing and Materials (ASTM) 3135 standards) were obtained, and the following experiments were conducted from April to August of 2023. Each of the three carriers along with three calibrated radiometers (ILT1270, International Light Technologies, Peabody, MA) were mounted on an aluminum stand at positions A (left of center of stand), B (center of stand), and C (right of center of stand). The stand was positioned at 9 feet (2.74 m) from and directly ahead of UV-C emitters utilizing low-pressure mercury gas UV-C lamps (Surfacide, Waukesha, WI). Five UV-C emitter configurations were assessed; (1) three emitters with a triangular configuration about the stand and each rotating 360 degrees, (2) one emitter facing the stand and rotating 360 degrees, (3) three emitters facing the stand in a linear configuration and each rotating 5 degrees, (4) one emitter facing the stand and rotating 5 degrees, and (5) one emitter facing the stand and rotating 90 degrees. Three serial experiments were conducted. The first experiment used a dose titration curve to identify the minimally effective irradiation dose (mean and standard deviation mJ/cm^2^) to achieve no growth (6-log reduction) in *C. difficile* counts with direct irradiation exposure. The second experiment involved assessing the relative efficacy of the five emitter configurations with the use of the minimally effective dose in attenuating polycarbonate *C. difficile* carriers positioned at 25.5 and 69.5 inches (64.77 to 176.53 cm) from the floor and oriented vertically to the emitters. The third experiment evaluated the relative efficacy of the five configurations for polycarbonate and textured plastic *C. difficile* carriers positioned at 25.5 or 58.5 inches (64.77 to 148.59 cm) from the floor and with a 45-degree or horizontal orientation to the emitters. We assessed residual anaerobic bacterial contamination for three intensive care unit (ICU) rooms to ascertain clinical applications of study results.

Results: The minimally effective dose for polycarbonate *C. diff*icile carriers with direct exposure was 432.28 ± 2.12 mJ/cm^2^. Configurations one through five achieved a > 4-log reduction when the minimally effective dose was delivered to polycarbonate *C. difficile* carriers that were positioned at 9 feet from the emitters, 25.5 or 69.5 inches from the floor, and with vertical orientation to the emitters. When *C. difficile* carriers were changed to textured plastic, orientation to the emitters was changed to horizontal or 45 degrees, and height from the floor was changed to 25.5 and 58.5 inches, the log reductions achieved by configuration one through five were 1.61, 0.61, 0.79, 1.15, and 0.98, respectively, with configuration one achieving a greater log reduction than two (P = 0.0137). In each of the three ICU rooms, at least one of nine sampled locations returned ≥ 500 anaerobic CFU, indicating the need for at least a 0.7-log reduction (500 to 99 CFU).

Conclusions: UV-C emitter configuration impacts efficacy in attenuating *C. difficile*.

## Introduction

Ultraviolet-C (UV-C) irradiation can reliably augment routine environmental cleaning procedures [1,2]. The previously reported impact on the incidence of healthcare-associated infections (HCAIs) is less clear [3-6]. Some variability in UV-C efficacy for HCAI prevention [3-6] may be driven by human factors that impact the use of the technology, such as concerns regarding disruption in patient care activities [7], but there are alternative explanations. For example, single measures are less effective than multimodal strategies for infection prevention. In a recent study, a multifaceted approach was associated with a 68% reduction in surgical site infections (SSIs) as compared to single interventions (risk ratio 0.32, 97.5% confidence interval 0.15-0.70, P = 0.001) [8]. Those study results [8] are supported by rigorous investigations of the epidemiology of bacterial transmission that have repeatedly shown that multiple reservoirs provide clinically relevant contributions to bacterial transmission events that subsequently lead to infection development [9,10]. Leveraging this earlier work [8-10], both a cluster randomized trial [11] and a large postimplementation analysis [12] showed that UV-C when incorporated as part of a multifaceted program can help to generate substantial reductions in bacterial transmission and surgical site infections [11,12]. Thus, future studies can help to establish more reliable outcomes with the use of UV-C by incorporating an evidence-based UV-C implementation strategy into a multifaceted infection control program [9-12]. The first step is the development of an evidence-based strategy for UV-C implementation.

Recent work has established that frequently touched environmental reservoirs in operating room [13] and intensive care unit (ICU) [14] environments that return ≥ 100 colony forming units (CFU)/surface area sampled are associated with an increased risk of major bacterial pathogen detection [13,14]. Furthermore, interventions that reduce environmental contamination below 100 CFU can reduce stopcock contamination [15] and infections [15,16] in operating room and ICU environments. As such, an evidence-based UV-C implementation strategy should be designed to reliably reduce residual environmental contamination below 100 CFU [13-16]. This should occur even in the setting of barriers to irradiation delivery such as treatment distance for a typical patient bay [17] and environmental target height from the floor, orientation to the emitters, and/or substrate [1].

In this study, we planned to leverage *Clostridioides difficile* (*C. difficile*), a clinically relevant [18-21] anaerobic pathogen that is particularly resistant to UV-C [22], to delineate an evidence-based UV-C implementation strategy. Our earlier work showing that a UV-C emitter configuration involving three emitters each rotating 360 degrees about the target was highly effective in attenuating the more pathogenic *Staphylococcus aureus* (*S. aureus*) sequence type 5 [23] led us to hypothesize that UV-C emitter configuration would impact attenuation of *C. difficile* carriers, especially in the setting of potential barriers to irradiation dose delivery. We aimed to evaluate the efficacy of five UV-C delivery systems that used low-pressure mercury gas lamps (Helios, Surfacide, Waukesha, WI). The configurations included a triangular configuration of three emitters about the target and each rotating 360 degrees, a linear configuration of three emitters facing the target and each rotating 5 degrees, and three variations involving the use of a single emitter (the status quo) [1], facing the target but with rotation ranging from 5 to 360 degrees. The five configurations tested in this study represent currently available multi [23] and single emitter [1] technology. We hypothesized that configuration one would be more efficacious than configuration two in attenuating *C. difficile *[23].

## Materials and methods

This was a laboratory-based and environmental study (RDB Bioinformatics) without patient involvement that was conducted in April through August of 2023. This was nonhuman subject research. This experimental design did not include human or animal subjects, tissue, or samples, so it was exempt from approval of the local ethics committee, thus there was no number assigned.

We anticipated attenuation of UV-C irradiation dose delivery due to planned assessment under conditions of indirect irradiation exposure (e.g., horizontal orientation to the emitters) in subsequent experiments. As such, we first established a dose of UV-C irradiation that could reliably achieve a > 6-log reduction (no growth) from baseline controls for directly exposed *C. difficile* carriers.

Commercially available *C. difficile* American Type Culture Collection (ATCC) 43593 polycarbonate carriers (ReadyNow^TM^ Test Carriers, Stratix Labs Corporation, Saint Paul, MN) were obtained. This technology facilitates reproducible use of a standard test method (American Society for Testing and Materials {ASTM} 3135: Standard Practice for Determining Antimicrobial Efficacy of Ultraviolet Germicidal Irradiation Against Microorganisms on Carriers with Simulated Soil) for assessment of disinfection efficacy [24]. Growth conditions for spore preparation include, per the manufacturer, inoculation of pre-reduced reinforced clostridial medium (RCM) (Thermo Fisher Scientific, Waltham, MA) with an isolated *C. difficile* colony followed by growth in an anaerobic chamber at 35+/-1°C for approximately 24 hours. The culture was then used to inoculate pre-reduced CDC anaerobic 5% sheep blood agar (CABA) plates (Anaerobe Systems, Morgan Hill, CA) that were incubated for approximately 10 days in an anaerobic chamber at 35+/-1°C. The spores were then harvested, and a coating solution with an initial inoculum >10^8^ CFU/mL was prepared by diluting or concentrating the spore suspension.

Approximately 1" x 0.9" polycarbonate slides were contaminated with ten, 10 µL drops of the stock solution (the soiling agent fetal bovine serum, FBS). Carriers were air-dried until the droplets were no longer visible, for 15-20 minutes [24]. The top of the cartridge was removable to allow exposure of the pre-inoculated carrier to disinfection agents, and the back side of the cartridge had an adhesive strip for mounting the device to vertical test surfaces. These carriers were used for both treatment and control conditions, and all carriers were prepared, handled, and processed for CFU enumeration using identical procedures as outlined below.

**Figure 1:**
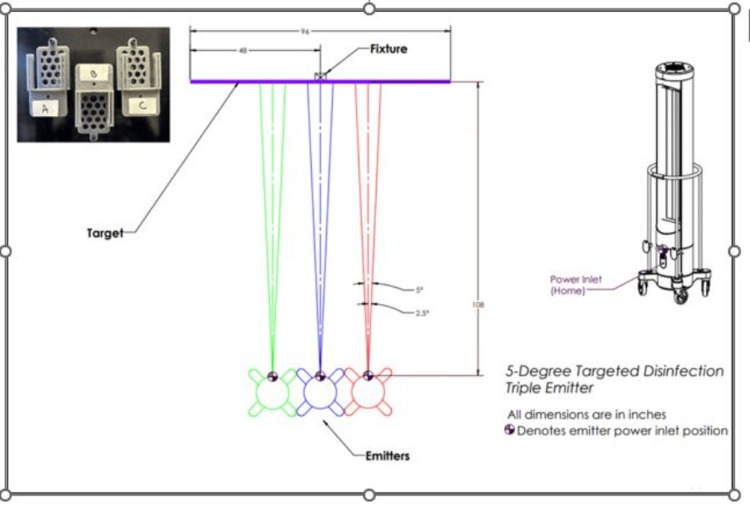
Ultraviolet-C (UV-C) dose response testing for Clostridioides difficile (C. difficile) American Type Culture Collection (ATCC) 43593 carriers. *C. difficile* carriers (1”x 0.9” polycarbonate, ReadyNow^TM^ Test Carriers, Stratix Labs Corporation, Saint Paul, MN) and calibrated radiometers (ILT1270, International Light Technologies, Peabody, MA) were mounted on an aluminum stand at three positions (A = center of stand, B = left of center of stand, C = right of center of stand) and at a height of 47.5 inches from the floor, center of bulbs. The stand was positioned at 9 feet from three low-pressure mercury gas UV-C emitters that were positioned in a row, each rotating 5 degrees. The test carriers and adjacent radiometers were exposed to an increasing dose of UV-C energy from 126.55-580.77 (mJ/cm^2^). Log reductions (LR) were calculated by comparing the average of treatment samples (final CFU) for a given dose to the average of positive controls (initial CFU), log_10 _(initial CFU/final CFU). Following treatment at each target dose, carrier and control slides were removed from the cartridge and processed identically to enumerate colony-forming units (CFU). Log reductions (LR) were calculated by comparing the average of treatment samples (final CFU) for a given dose to the average of positive controls (initial CFU), log_10 _(initial CFU/final CFU). This image was created by the authors.

The laboratory testing area was confirmed to be free from line-of-sight obstructions. Three UV-C emitters using low-pressure mercury gas lamps were positioned in a row facing an aluminum stand placed 9 feet (2.74 m) from the emitters. Distance to the center of the stand was measured via the use of a calibrated tape measure from the blue power inlet on the center of the middle emitter. Nine feet estimate the effect to be generated for whole room disinfection involving a target disinfection width of 8 feet (2.43 m), or coverage of a typical patient bay [17]. Each of the three pathogen carriers and previously calibrated radiometers (ILT1270, International Light Technologies, Peabody, MA) were mounted on the stand at positions A (left of center of stand), B (center of stand), and C (right of center of stand), facing the fixtures and positioned directly ahead of the center emitter (Figure 1).

To measure the delivered radiation, the radiometers were positioned vertically to the emitters. As the highest irradiance is generated from the center of the bulbs, carriers, and radiometers were placed at the center of the bulbs measured at 47.5 inches (120.65 cm) from the floor, and the radiometers were calibrated to 254-nm irradiation, the peak intensity of UV-C. Irradiance, W/cm^2^, or power/cm^2^, was measured by the radiometers. The delivered dose was the time of irradiance exposure, W/cm^2^ X time (seconds) of exposure, or J/cm^2^. UV-C emitters were allowed to warm up for 10 minutes outside of the test room and returned to the marked locations. Three emitters positioned side-by-side, each rotating five degrees, delivered energy to the carriers at increasing doses.

The test carriers and adjacent radiometers were exposed to an increasing dose of UV-C energy. Radiometers were connected and the software opened sequentially with display units of J/cm^2^. As emitters were turned off after all three emitters had reached the target incremental dose, the average max cumulative dose for the three radiometers was calculated and recorded.

Following treatment at each target dose, the ATCC carrier polycarbonate slides were removed from the cartridge and placed into a 50 mL conical tube (Thermo Fisher Scientific, Waltham, MA) containing 10 mL, 10,000 µl, of phosphate-buffered saline (PBS) (Thermo Fisher Scientific, Waltham, MA), a 10^-3^ dilution. Conical tubes were vortexed on high for 30 seconds, and serial dilutions were made as follows: 10 µl of solution to 990 µl of PBS and five seconds of vortexing on high, 10^-6^, and 100 µl of the 10^-6^ solution to 900 µl of PBS, 10^-7^. Each dilution (100 µL) was then plated to brain heart infusion agar with horse blood and taurocholate (Anaerobe Systems AS6463, Thermo Fisher Scientific, Waltham, MA). The plates were then placed into anaerobic pouches (BD GasPak EZ anaerobe pouch system, Franklin Lakes, NJ) and incubated at 36.5°C for 24 hours. Total CFU was quantified at 24 hours, where ≥ 500 CFU were considered too numerous to count and recorded as 500 [11,12]. Log reductions (LR) were calculated by comparing the average of treatment samples (final CFU) for a given dose to the average of positive controls (initial CFU), log_10 _(initial CFU/final CFU).

Next, we assessed the efficacy of delivery of the minimally effective irradiation dose via five different emitter configurations along with variations in carrier height from the floor. *C. difficile* carriers were positioned on a stand 9 feet from and vertically oriented to the various emitter configurations and at 25.5 and 69.5 inches (64.77 and 176.53 cm) from the floor.

**Figure 2:**
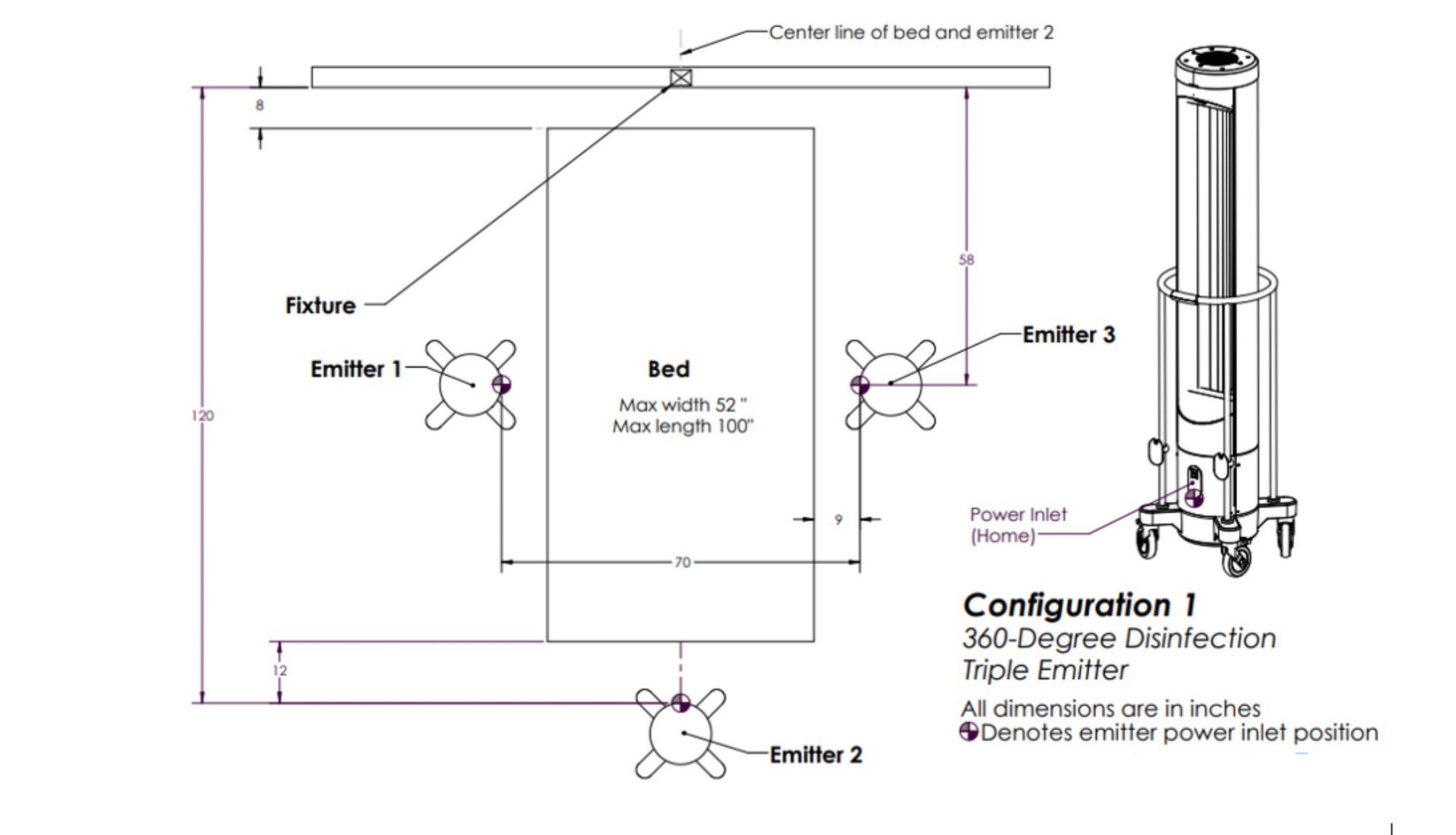
Ultraviolet-C (UV-C) emitter configuration one. Three emitters positioned triangularly about the target and each rotating 360 degrees. This image was created by the authors.

Emitter one involved three emitters each rotating 360 degrees positioned triangularly about the target (Figure 2).

**Figure 3:**
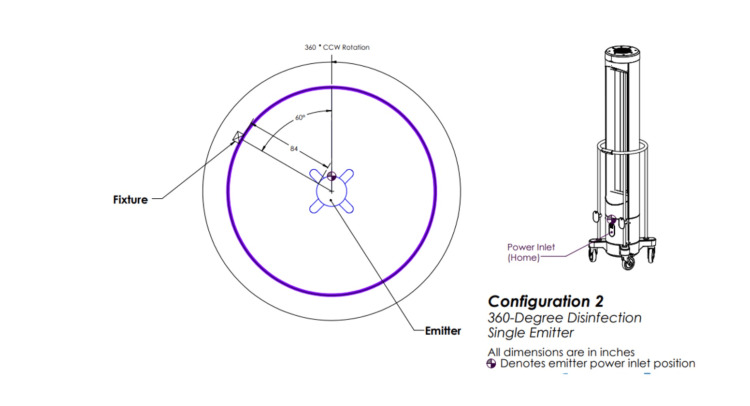
Ultraviolet-C (UV-C) emitter configuration two. One emitter positioned in a room and rotating 360 degrees. This image was created by the authors.

Emitter two involved one emitter rotating 360 degrees (Figure 3).

**Figure 4:**
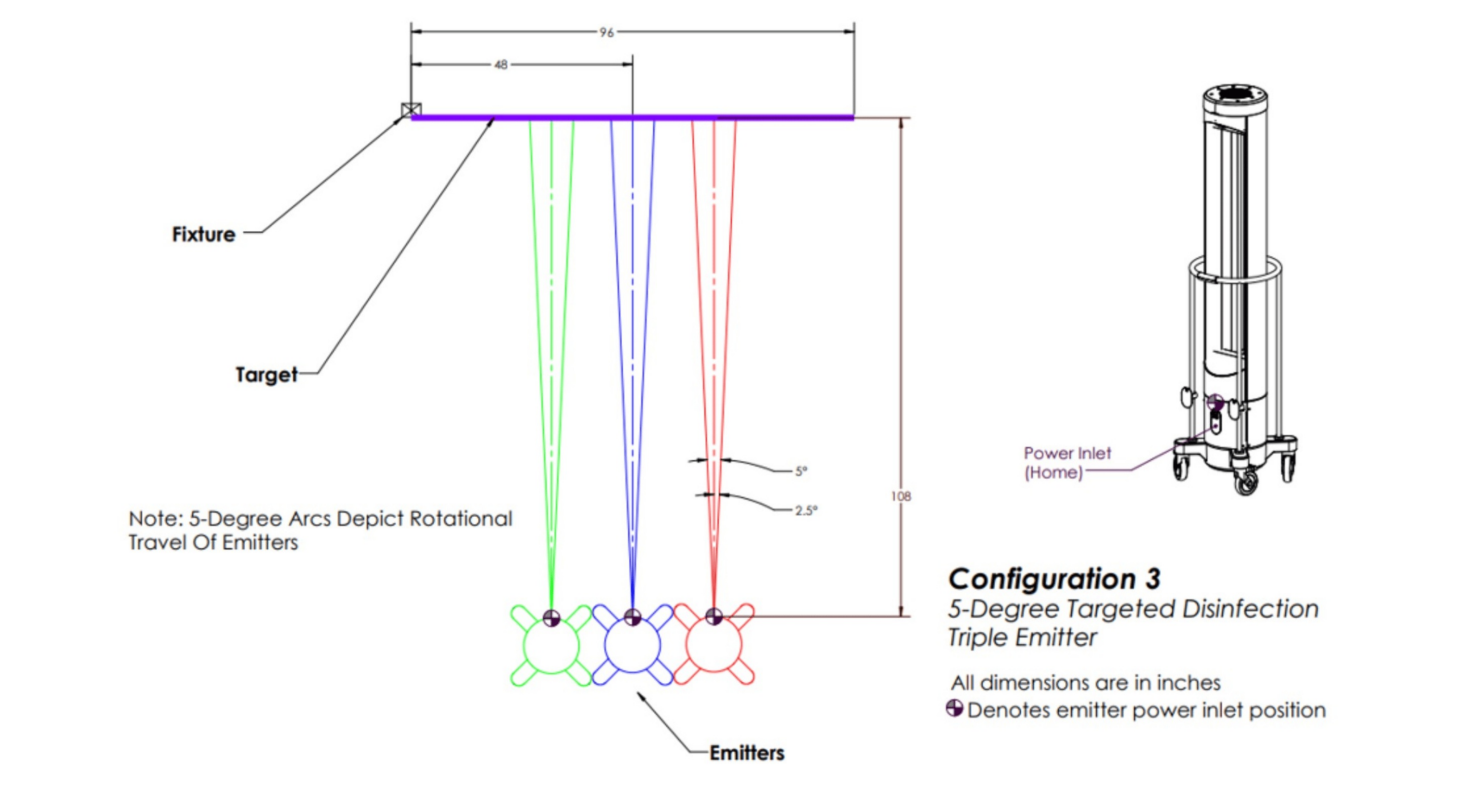
Ultraviolet-C (UV-C) emitter configuration three. Three emitters positioned in front of the target in a row and each rotating 5 degrees. This image was created by the authors.

Configuration three involved three emitters positioned in a row in front of the target, each rotating five degrees (Figure 4).

**Figure 5:**
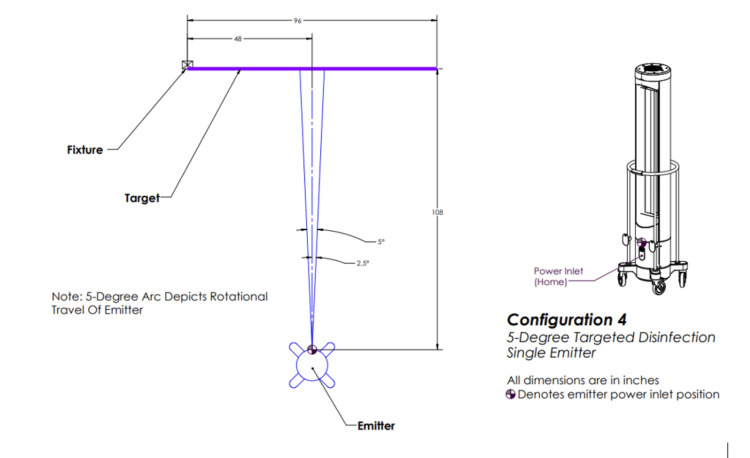
Ultraviolet-C (UV-C) emitter configuration four. One emitter positioned in front of the target and rotating 5 degrees. This image was created by the authors.

Configuration four involved one emitter positioned in front of the target and rotating five degrees (Figure 5).

**Figure 6:**
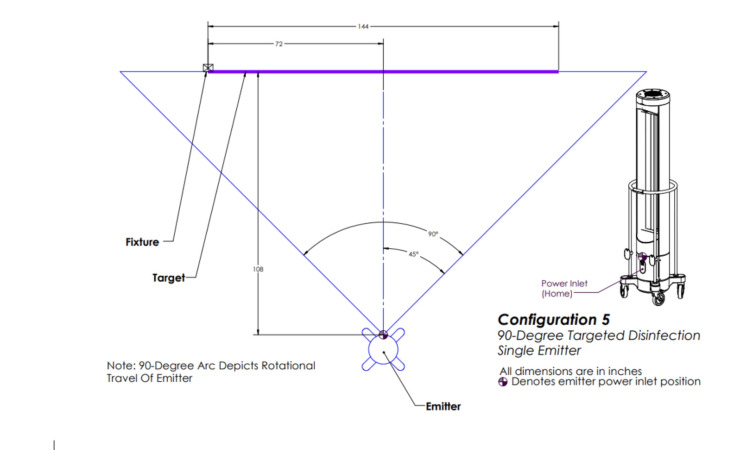
Ultraviolet-C (UV-C) emitter configuration five. One emitter positioned in front of the target and rotating 90 degrees. This image was created by the authors.

Configuration five involved one emitter positioned in front of the target and rotating 90 degrees (Figure 6).

Soiling involved FBS. Each carrier was immediately processed as described above, CFU quantified, and LR calculated.

We then assessed the relative efficacy of the five configurations above while varying substrate material, height from the floor, and orientation to the emitters. We chose 58.5 inches (148.59 cm) from the floor for this set of experiments because 58.5 inches is midrange between the center (47.5 in) and top (69.5 in) of the lamp, allowing an incremental assessment of height from the floor, or the impact of the angle of incidence on efficacy. We evaluated textured acrylonitrile butadiene styrene (ABS) plastic vs. polycarbonate carriers (ReadyNow^TM^ Test Carriers, Stratix Labs Corporation, Saint Paul, MN) and horizontal and 45-degree orientation of carriers to the emitters.

Additional assessments included the determination of the following: 1) We assessed the cycle time required for dose delivery throughout the disinfection space (height and distance) for the applicable equipment configuration, lamp warm-up time (10 minutes), and emitter to emitter variation was determined, 2) We evaluated the relative dose delivery to horizontal and vertical target surfaces. Using configuration three at 9 feet and at 25.5, 47.5, and 58.5 inches from the floor, we simultaneously measured delivered irradiance (mJ/cm^2^) to radiometers oriented vertically and horizontally to the emitters until horizontal emitters reached the minimally effective dose of 432 mJ/cm^2^, and 3) We examined the efficacy of delivery of UV-C irradiance of 300-600 mJ/cm^2^ to *C. difficile* carriers when oriented horizontally to the emitters.

Finally, to judge how to apply the results clinically, we assessed the current residual contamination with anaerobes. We identified three ICU rooms at the University of Iowa that were at least 8 feet wide and 9 feet deep, had undergone terminal cleaning, and were ready for patient admission on August 8, 2023. Rooms included a private room in the surgical neuro intensive care unit bay two, a shared room in the surgical neurosciences intensive care unit bay three, and a private room in the cardiovascular intensive care unit. Surface disinfection involved the use of a quaternary ammonium compound according to the usual protocol, and the rooms were ready for patient occupancy. Twenty-seven samples (nine for each of the three rooms) were obtained from a variety of surfaces and equipment materials at various heights and with various orientations to the emitters after surface disinfection. These sample locations included the door handle, bedside tabletop, bedside table side, bedrail top, bedrail front, medication pump front, medication pump side, computer screen, and desk. Because some sites were irregular in shape [e.g., door handle, medication pump (buttons and ridges), and tabletop side (lip)], samples were collected using a dry ESwab (Copan, Murrieta, CA) [25]. If the sampled area was < 10x10 cm^2^, the entire surface area was sampled. If the sampled area was > 10x10 cm^2^, a 10x10 cm^2^ area was sampled [25]. The samples were sent to the lab immediately, vortexed for five seconds on medium-high, 1:100 dilutions made, 100 µl of each 1:100 dilution plated to sheep’s blood agar (SBA) (Thermo Fisher Scientific, Waltham, MA), and the SBA plates incubated at 36°C for 24 hours under aerobic conditions. CFU was quantified. Samples with aerobic growth were re-plated under anaerobic conditions [26].

Statistical analyses were the reporting of the mean, maximum, and cumulative dose and associated log reductions along with CFU mean/SD using simple descriptive statistics. Wilcoxon-Mann-Whitney was used to compare configurations one and two, with two-sided exact P <0.05 treated as statistically significant (Stata v18.5, StataCorp, College Station, TX).

## Results

The minimally effective dose required to achieve a > 6-log reduction for ≥ two consecutive treatments of *C. difficile* carriers positioned at 9 feet and at the center of the lamp was 432.28 ± 2.12 mJ/cm^2 ^(Table 1).

**Table 1:** Ultraviolet-C (UV-C) dose response curve for Clostridioides difficile, the dose being shown in the first column and the response shown in the fifth column. The average max cumulative dose is for the three radiometers in positions A, B, and C; SD = standard deviation; average positive control colony-forming units (CFU)/mL is the average of CFU for each position A, B, and C where each position involved two dilutions (N=6 for the mean), which also applies to the average UV-C treatment CFU/mL. The same control CFU indicates the same experiment. Log reduction uses base 10 (e.g., 2.25 = log_10 _(1.47 X 10^8^/8.33 x 10^5^)).

Average max cumulative dose (mJ/cm^2^)	SD	Average positive control CFU/mL	Average UV-C treatment CFU/mL	Log reduction
126.55	0.58	1.47 x 10^8^	8.33 x 10^5^	2.25
178.35	1.13	1.47 x 10^8^	0	> 6
228.39	1.31	1.47 x 10^8^	1.67 x 10^6^	1.94
279.77	1.36	1.47 x 10^8^	0	> 6
345.81	0.98	1.47 x 10^8^	1.67 x 10^5^	2.94
379.55	1.82	1.47 x 10^8^	1.67 x 10^5^	2.94
432.28	2.12	1.47 x 10^8^	0	> 6
480.85	2.89	1.47 x 10^8^	0	> 6
530.10	3.07	1.47 x 10^8^	0	> 6
580.77	3.04	1.47 x 10^8^	0	> 6

Delivery configurations one through four achieved a > 6-log reduction and configuration five a 4-log reduction with the delivery of the minimally effective dose to polycarbonate carriers with vertical orientation to the emitters and at 25.5 and 69.5 in from the floor (Table 2).

**Table 2:** Impact of ultraviolet-C (UV-C) delivery configuration and height on Clostridioides difficile attenuation Configuration one (three towers positioned triangularly each rotating 360 degrees), configuration two (one tower rotating 360 degrees), configuration three (three towers in a line, each rotating five degrees), configuration four (one tower rotating five degrees), and configuration five (one emitter rotating 90 degrees). All carriers were oriented vertically to the emitters, mJ/cm^2^ was the dose of irradiation delivered to the carriers at 25.5 or 69.5 inches from the floor. SD = standard deviation, positive ctrl (control) was the average of the colony-forming units (CFU) for each of two dilutions (10^-6^ and 10^-7^) for each of the three carrier positions (A, B, and C) on the aluminum stand positioned in front of the emitters at a distance of 9 feet (initial CFU), N=6 for the average. The same applies to UV-C CFU/mL at 25.5 and 69.5 inches (final CFU).  LR = log reduction defined by log_10 _(initial CFU/final CFU). For example, log_10_ (3.25 x 10^7^/3.33 x 10^5^) = 1.99. The average LR refers to the average LR for the two heights at a given dose, for example, 1.99 + 6/2 = 4.

Config	25.5 in mJ/cm^2^	SD 25.5 in mJ/cm^2^	69.5 in mJ/cm^2^	SD 69.5 in mJ/cm^2^	Positive Ctrl CFU/mL	UV-C 25.5 in CFU/mL	LR	UV-C 69.5 in CFU/mL	LR 69.5 in	Average LR 25.5 and 69.5 in
One	447.91	28.56	440.83	18.69	3.47 x 10^7^	0	>6	0	>6	>6
Two	446.10	16.92	430.38	4.28	1.30 x 10^8^	0	>6	0	>6	>6
Three	442.01	16.99	449.66	21.65	1.11 x 10^8^	0	>6	0	>6	>6
Four	448.75	24.31	444.33	16.87	1.54 x 10^8^	0	>6	0	>6	>6
Five	444.41	19.95	434.38	14.53	3.25 x 10^7^	0	>6	3.33 x 10^5^	1.99	4

The average log reduction for configurations one through five when textured plastic carriers were positioned at 9 feet, oriented 45 degrees or horizontally to the emitters, and at 25.5 or 58.5 in from the floor was 1.19 ± 1.24 mJ/cm^2^. The respective log reductions for configurations one through five were 1.61, 0.61, 0.79, 1.15, and 0.98, respectively (Table 3).

**Table 3:** With the target textured plastic, the impact of ultraviolet-C (UV-C) configuration and target orientation, position, and height on Clostridioides difficile attenuation. Configuration one (three towers positioned triangularly each rotating 360°), configuration two (one tower rotating 360°), configuration three (three towers in a line, each rotating 5°), configuration four (one tower rotating 5°), and configuration five (one emitter rotating 90°), positions one through seven were different positions selected within range of configuration one. Orientation refers to how the target *C. difficile* carriers were positioned relative to the emitter(s), either facing directly (vertical) or horizontal, height is from the floor, mJ/cm^2^ is the average dose of irradiance recorded by radiometers positioned at A, B, and C about the stand where the *C. difficile* carriers were affixed. Ctrl = control, CFU = colony-forming units, ctrl CFU is the average of the positive control CFU for positions A, B, and C where each position involved two dilutions, where the number is the same indicates the same experiment and the same set of controls, N=six for the average. The same applies to UV-C CFU, LR = log reduction.

Configuration	Position	Orientation	Height (in)	mJ/cm^2^	Ctrl CFU	UV-C CFU	LR
One	One	Horizontal	47.5	435.35	6.97 x 10^7^	1.47 x 10^7^	0.68
One	One	45 degrees	47.5	436.72	6.97 x 10^7^	1.17 x 10^6^	1.78
One	One	45 degrees	58.5	435.77	6.97 x 10^7^	1.17 x 10^6^	1.78
One	One	Horizontal	58.5	433.27	6.97 x 10^7^	1.17 x 10^6^	1.78
One	Two	Horizontal	58.5	442.47	6.97 x 10^7^	9.57 x 10^7^	0
One	Two	45 degrees	58.5	436.08	6.97 x 10^7^	5.67 x 10^6^	1.09
One	Two	45 degrees	47.5	441.03	6.97 x 10^7^	0	> 6
One	Two	Horizontal	47.5	436.83	6.97 x 10^7^	5.80 x 10^7^	0.08
One	Three	Horizontal	58.5	438.44	1.17 x 10^8^	4.15 x 10^7^	0.45
One	Three	45 degrees	58.5	437.94	1.17 x 10^8^	1.17 x 10^6^	2
One	Three	45 degrees	47.5	435.72	1.17 x 10^8^	1.67 x 10^5^	2.85
One	Three	Horizontal	47.5	433.55	1.17 x 10^8^	1.43 x 10^7^	0.91
One	Four	Horizontal	47.5	441.37	1.17 x 10^8^	1.29 x 10^8^	0
One	Four	45 degrees	47.5	458.02	1.17 x 10^8^	5.0 x 10^5^	2.37
One	Four	45 degrees	58.5	450.21	1.17 x 10^8^	2.00 x 10^6^	1.77
One	Four	Horizontal	58.5	451.58	1.17 x 10^8^	3.85 x 10^7^	0.48
One	Five	Horizontal	58.5	431.07	1.17 x 10^8^	5.27 x 10^7^	0.35
One	Five	45 degrees	58.5	432.63	1.17 x 10^8^	3.00 x 10^6^	1.59
One	Five	Horizontal	47.5	434.81	1.17 x 10^8^	3.08 x 10^7^	0.58
One	Five	45 degrees	47.5	432.32	1.17 x 10^8^	1.17 x 10^6^	2
One	Six	Horizontal	47.5	434.27	1.17 x 10^8^	9.9 x 10^7^	0.07
One	Six	45 degrees	47.5	433.13	1.17 x 10^8^	1.17 x 10^6^	2
One	Six	45 degrees	58.5	435.8	1.17 x 10^8^	1.00 x 10^6^	2.07
One	Six	Horizontal	58.5	435.53	1.17 x 10^8^	3.10 x 10^7^	0.58
One	Seven	45 degrees	47.5	430.84	1.17 x 10^8^	3.33 x 10^5^	2.55
One	Seven	Horizontal	47.5	431.25	1.17 x 10^8^	0	> 6
Two	N/A	Horizontal	47.5	432.79	5.34 x 10^7^	1.11 x 10^8^	0
Two	N/A	45 degrees	47.5	431.06	5.34 x 10^7^	8.33 x 10^6^	0.81
Two	N/A	45 degrees	58.5	428.56	5.34 x 10^7^	3.17 x 10^6^	1.23
Two	N/A	Horizontal	58.5	431.42	5.34 x 10^7^	2.63 x 10^7^	0.31
Two	N/A	Horizontal	25.5	434.26	3.18 x 10^7^	1.15 x 10^7^	0.44
Two	N/A	Horizontal	47.5	430.56	3.18 x 10^7^	7.70 x 10^7^	0
Two	N/A	Horizontal	58.5	431.26	3.18 x 10^7^	1.63 x 10^8^	0
Two	N/A	Horizontal	47.5	431.77	4.48 x 10^7^	1.08 x 10^7^	0.62
Two	N/A	45 degrees	47.5	433.22	4.48 x 10^7^	1.17 x 10^6^	1.58
Two	N/A	45 degrees	58.5	429.15	4.48 x 10^7^	8.33 x 10^5^	1.73
Two	N/A	Horizontal	58.5	430.22	4.48 x 10^7^	3.03 x 10^7^	0.17
Two	N/A	Horizontal	25.5	427.56	8.07 x 10^7^	1.58 x 10^7^	0.71
Two	N/A	Horizontal	47.5	430.84	8.07 x 10^7^	2.08 x 10^7^	0.59
Two	N/A	Horizontal	58.5	428	8.07 x 10^7^	3.48 x 10^7^	0.37
Three	N/A	Horizontal	58.5	448.55	5.60 x 10^7^	2.57 x 10^7^	0.34
Three	N/A	45 Degrees	58.5	450.32	5.60 x 10^7^	3.50 x 10^6^	1.2
Three	N/A	45 Degrees	47.5	446.31	5.6 x 10^7^	3.67 x 10^6^	1.18
Three	N/A	Horizontal	47.5	445.13	5.60x 10^7^	2.15 x 10^7^	0.42
Four	N/A	Horizontal	47.5	455.98	4.68 x 10^7^	1.17 x 10^7^	0.6
Four	N/A	45 Degrees	47.5	454.24	4.68 x 10^7^	5.00 x 10^5^	1.97
Four	N/A	45 Degrees	58.5	455.79	4.68 x 10^7^	8.33 x 10^5^	1.75
Four	N/A	Horizontal	58.5	455.15	4.68 x 10^7^	2.47 x 10^7^	0.28
Five	N/A	Horizontal	58.5	438.5	2.24 x 10^8^	9.57 x 10^7^	0.37
Five	N/A	45 Degrees	58.5	438.45	2.24 x 10^8^	5.00 x 10^6^	1.65
Five	N/A	45 Degrees	47.5	439	2.24 x 10^8^	4.83 x 10^6^	1.67
Five	N/A	Horizontal	47.5	438.58	2.24 x 10^8^	1.29 x 10^8^	0.24

The log reduction achieved by configuration one was higher than that achieved by configuration two (P = 0.0137).

Treatment times required for delivery of the target dose of 432 mJ/cm^2 ^for configurations one through five were 30, 53, 8, 21, and 43 minutes, respectively. Delivery of 432 mJ/cm^2^ to radiometers oriented horizontally was associated with a mean delivery of 20.93 ± 17.55 J/cm^2^ to vertical radiometers at 25.5 to 58.5 in from the floor (Table 4).

**Table 4:** Configuration three horizontal irradiance. Configuration three - three emitters in a row, each rotating five degrees, target dose - 432 mJ/cm^2^ delivered to a space 8 feet wide and 9 feet deep, horizontal dose - measured irradiance by the horizontal radiometer, vertical dose - measured irradiance by the vertical radiometer.

Radiometer position	Target dose	Horizontal dose	Vertical dose
25.5” horizontal	432 mJ/cm^2^	432 mJ/cm^2^	4, 706 mJ/cm^2^
47.5” horizontal	432 mJ/cm^2^	432 mJ/cm^2^	18.53 J/cm^2^
58.5” horizontal	432 mJ/cm^2^	432 mJ/cm^2^	39.56 J/cm^2^

The log reduction for ≥ 300 mJ/cm^2^ of irradiance delivered to *C. difficile* carriers oriented horizontally to the emitters was > 6.

The three ICU rooms each had nine frequent locations swabbed and cultured. There were four positive anaerobic cultures that included the front of the bedside table, the top of the bedrail, the front of the bedside table, and the front of the medication pump. The four positive cultures all had ≥ 500 colony-forming units, the dilutions too numerous to count.

## Discussion

The impact of UV-C emitter configuration on attenuation of target pathogens has not been previously assessed. In this study, we assessed the relative efficacy of five different UV-C emitter configurations that represent commercially available devices along with variations in target height, orientation, and substrate. We focused on *C. difficile* given its resistance to UV-C irradiation and clinical relevance [18-22]. We show that UV-C delivery configuration is an important consideration for UV-C implementation and/or evaluation of efficacy for HCAI prevention.

Prior work in the clinical environment has assessed the efficacy of a variety of UV-C devices for HCAI prevention [1-6]. Limitations of this prior work included the use of only one delivery configuration, a single emitter rotating 360 degrees, and configuration two in this experiment. More recent work assessed a triangular configuration of three emitters, configuration one in this experiment, and found that there was significant attenuation of the more pathogenic *S. aureus* sequence type five despite barriers to dose delivery such as horizontal orientation to the emitters [23]. This same configuration was shown previously in a cluster randomized trial [11] and in a large postimplementation analysis [12] to generate substantial reductions in *S. aureus* transmission and 90-day postoperative surgical site infections when included in a multifaceted infection control program [11,12]. This prior work [11,12,23] led us to hypothesize that the position of UV-C emitters during irradiation delivery to environmental surfaces may impact efficacy for pathogen attenuation and subsequent HCAI development. We hypothesized that configuration one would be more efficacious than configuration two, the status quo. There were however limitations of prior work extending beyond the use of a single emitter configuration. Laboratory assessments of UV-C were conducted at distances of less than 9 feet [27], thereby failing to consider the potential impact of the treatment distance required for a typical patient bay, 9 feet [17]. This is a significant limitation, as distance is inversely related to dose delivery (decreased dose delivery with increasing distance from the emitter). While prior work has considered the impact of target height from the floor, material, and orientation to the emitters [27], these factors were not considered in parallel with a distance that would be faced clinically [17]. Impaired dose delivery is not likely to be accounted for by simply moving an emitter closer to an object (whether robotic or manual), as movement towards one object is inherently away from another. Both objects could be contaminated with an invisible pathogen. Furthermore, near-field over-treatment could potentially result in photo reactivation, dark repair, and subsequent regrowth [28]. An alternative approach includes whole-room treatment where room area and target pathogens are important considerations. Prior work also did not confirm that residual CFU was reduced to < 100 CFU [13,14]. This standard for surface hygiene [13,14] has been recently discovered in the clinical arena despite being well-grounded in the food industry [25]. Taken together, these limitations suggest that the irradiation energy released from the emitters employed for prior clinical studies may not have been effectively delivered to the targets due to distance, emitter configuration, and/or barriers to dose delivery [1-6]. Alternatively, the energy delivered may have been insufficient to attenuate the target pathogen(s) [1] or too high resulting in subsequent regrowth [28]. Further, UV-C was tested as a single modality when evidence suggests that a multimodal approach is indicated [8-12]. Thus, current gaps regarding the use of UV-C for HCAI prevention are several and include the need for the development of an evidence-based implementation strategy for UV-C as part of a multifaceted program.

In this study, we add to the current body of literature by testing the hypothesis that emitter positioning about a target, emitter configuration, can impact UV-C efficacy. We established a minimally effective dose for achieving a 6-log reduction in *C. difficile* under direct exposure to the center of the lamp. This was an important first step, as our goal was to deliver a dose that could be measured in later experiments despite attenuation of delivery due to indirect exposure. In parallel, we planned to avoid over-treatment, photoreactivation dark repair, and subsequent regrowth [28]. In a stepwise fashion, we evaluated the relative efficacy of delivery of the minimally effective dose via five different delivery configurations that represent clinically available options. Most technology employs a single emitter rotating 360 degrees, configuration two in this study [1]. Our evaluations were at 9 feet [17], and we varied target height from the floor, orientation to the emitters, and substrate. Configuration one achieved a higher log reduction from baseline controls than configuration two despite potential barriers. Configuration two had the lowest observed performance with a 0.61 log reduction and treatment time of 43 minutes.

Thus, in this study, three emitters triangularly positioned about the target (configuration one) outperformed an emitter configuration that is typically used in practice for attenuation of *C. difficile *(configuration two)* *[1] despite barriers. These results are consistent with the reliable performance of configuration one in recent studies [11,12,23]. The enhanced efficacy of configuration one has face validity. The triangular positioning of the target increases the treatment area which may more effectively address shadowing. While we did not directly address shadowing in the clinical environment, we evaluated height from the floor and horizontal orientation. These assessments address the conceptual framework of shadowing because a shadow occurs when there is a deficiency in light exposure, and factors such as horizontal orientation limit light exposure. Additionally, the triangular configuration emits a more evenly distributed and lower dose of irradiation vs. a linear configuration [23]. This may reduce photoreactivation and dark repair [23,28]. As we show in this study, with the delivery of the minimally effective dose to horizontal surfaces via the use of the liner configuration three and increased time, there is substantial exposure of vertical surfaces, approximately 19-40 J/cm^2^. This is an alarmingly high dose given that 2 mJ/cm^2 ^was previously shown to sufficiently attenuate an *S. aureus* strain characteristic with increased strength of biofilm formation and desiccation tolerance [23] and 432 mJ/cm^2 ^is sufficient for *C. difficile, *a pathogen with intrinsic UV-C resistance [21], under conditions of direct exposure. It is likely that surfaces receive such doses when one simply moves an emitter closer to a target object. Furthermore, the triangular configuration is practical, as one emitter can be positioned at the foot of the bed, on one side of the bed, which is likely to be near the bathroom door, and one at the other side of the bed which is likely to be near monitors. Such positioning provides ample opportunity for whole-room disinfection. Furthermore, the treatment time for whole room disinfection only requires 30 minutes with configuration one, an acceptable duration [1].

Consideration of the relevance of the reported log reductions can help to further characterize the importance of emitter configuration. Residual contamination of frequently touched sites in the ICU should be assessed via the use of swabs for irregular surfaces and contact plates for flat surfaces [25]. Unfortunately, most studies have used contact plates for both irregular (e.g., a door handle) and flat (e.g., bed rail) surfaces [29], which likely underestimates the true magnitude of contamination. Not only are plates unable to capture the irregular surface area, but they are also less effective than swabs for capturing the 20% of pathogens that are more likely to contribute to cross-contamination [25]. A more recent study that appropriately used swabs to culture frequently touched ICU surfaces in the ICU [14] showed that 20% and 60% of sites exceeded 100 CFU by 12 and 24 hours, respectively, following active decontamination with a quaternary ammonium compound. As this threshold is associated with an increased risk of major bacterial pathogen detection in the OR [13] and in the ICU [14], and when environmental contamination is reduced below 100 CFU HCAIs fall [8-12], this degree of environmental contamination is clinically relevant. A minimal standard for surface hygiene in the clinical arena should therefore be to ensure that samples return less than 100 CFU [8-12], consistent with a food industry standard [25]. Prior studies did not assess anaerobic contamination [8-12]. We observed residual anaerobic contamination following routine surface disinfection cleaning of frequently touched sites in three different ICU environments. Each of the three rooms had at least one site with residual contamination that returned ≥ 500 CFU. While more reservoir observations among ICU rooms are needed to apply mathematically both the mean and the standard deviation of CFU [30], our results suggest that at least a 0.7-log reduction (reducing at least 500 CFU to 99 CFU) is indicated. We consider a 0.7-log reduction to represent the minimal desired effect of UV-C technology, where a 2-3-log reduction (500 to 0) would be more desirable. 

Understanding the minimal desired effect can help to interpret the study results. We found that all configurations but configuration two, a single emitter rotating 360 degrees, can achieve an observed 0.7-log reduction when there are substantial barriers to dose delivery. As most studies have used configuration two, this finding can help to explain in part variability in UV-C efficacy [1-6]. If there is substantial contamination, barriers, and/or surface area, the use of configuration two is unlikely to generate substantial pathogen attenuation, especially for the more resistant, spore-forming *C. difficile *[20,21]. Given that configuration can impact UV-C efficacy, these data suggest that the use of UV-C is not as simple as putting a device in a room and letting it spin according to manufacturer recommendations. We suggest that institutions and infection control officers use an evidence-based approach to deployment. This involves the following: 1) monitoring target environmental contamination levels, 2) using evidence for a standard of surface hygiene to identify treatment goals based on starting contamination levels (reducing starting contamination to a minimum of less than 100 CFU per surface area sampled [13,14], and 3) integrating knowledge of the target room area and barriers to dose delivery in deciding the planned UV-C approach, including but not limited to choice of UV-C emitter configuration (mode of deployment). Practically, if contamination is relatively high, there is a large surface area, and/or there is a significant risk of shadowing, configuration one is likely to be the better option. Configuration two, a single emitter rotating 360 degrees in a room, is a less desirable option. Future studies should incorporate this pragmatic UV-C implementation strategy into a multifaceted approach [8-12] and evaluate the impact on *C. difficile* cross-contamination and subsequent infections.

Thus, the main findings of this study suggest that UV-C emitter configuration is an important consideration that can impact the efficacy for attenuation of *C. difficile*. We show that a common configuration used in practice, a single emitter rotating 360 degrees (configuration two), had the lowest observed performance, whereas three emitters with a triangular position about the target (configuration one) had the highest observed performance. Importantly, the status quo failed to meet the minimum for a clinically relevant log reduction of 0.7 in the setting of barriers to irradiation dose delivery, which is as we describe, a benchmark for minimal performance. Future UV-C implementation for infection prevention and/or evaluation of efficacy should strongly consider extending beyond the use of a single emitter rotating 360 degrees.

A laboratory focus is one study limitation. Our laboratory evaluation did not directly assess the impact of UV-C on shadowed areas in the clinical environment. However, our experimental design accounted for shadowing given the assessment of the impact of horizontal orientation, extreme heights, and different materials at distance, factors that attenuate dose delivery; a shadow occurs when there is attenuation of light (dose) delivery. Additionally, this laboratory analysis can serve as the foundation for future clinical trials designed to further solidify these results. We evaluated *C. difficile* in this study, but we have previously shown that the high-performing configuration one is highly effective against multiple *S. aureus* sequence types frequently encountered among anesthesia workspace reservoirs [11,12,23]. For generalizability, we tested variations in emitter configurations that include currently available technology [1,23], and our study involved commonly employed low-pressure mercury gas UV-C lamps. We examined the impact of the delivery of an irradiation dose successfully in generating no growth under conditions involving direct pathogen carrier exposure. We were not testing the efficacy of irradiation. We were evaluating the relative efficacy of modes of irradiation delivery. Thus, with control of the delivered dose across multiple different technologies, the results of this study are generalizable and practical. The reproducible, standardized approach [24] used for our experimental design can be leveraged in future studies to compare log reductions for different UV-C technologies, but that was not the aim of this study. Instead, our aim was to help determine an evidence-based delivery mode for whatever technology is employed. This study was about the optimal use of a given UV-C technology. There was a 10-minute warm-up time for the device tested in this study, but this would not necessarily increase treatment time because repeat warm-up is not required with sustained use of the device.

## Conclusions

UV-C emitter configuration can impact attenuation of *C. difficile*. This parameter should be carefully considered for future clinical applications and/or study of UV-C technology, including those designed to reduce *C. difficile* cross contamination and subsequent infections in the ICU environment. Observation of anaerobic contamination among multiple ICUs and hospitals can guide dosing decisions.
